# Impact of the choice of reference genome on the ability of the core genome SNV methodology to distinguish strains of *Salmonella enterica* serovar Heidelberg

**DOI:** 10.1371/journal.pone.0192233

**Published:** 2018-02-05

**Authors:** Valentine Usongo, Chrystal Berry, Khadidja Yousfi, Florence Doualla-Bell, Genevieve Labbé, Roger Johnson, Eric Fournier, Celine Nadon, Lawrence Goodridge, Sadjia Bekal

**Affiliations:** 1 Laboratoire de Santé Publique du Québec, Sainte-Anne-de-Bellevue, Québec, Canada; 2 Department of Food Science and Agricultural Chemistry, Food Safety and Quality Program, McGill University, Ste Anne de Bellevue, Québec, Canada; 3 Division of Enteric Diseases, National Microbiology Laboratory, Public Health Agency of Canada, Winnipeg, Manitoba, Canada; 4 National Microbiology Laboratory at Guelph, Public Health Agency of Canada, Guelph, Ontario, Canada; 5 Département de Microbiologie, Infectiologie et Immunologie, Université de Montréal, Montréal, Québec, Canada; Laboratoire National de Santé, LUXEMBOURG

## Abstract

*Salmonella enterica* serovar Heidelberg (*S*. Heidelberg) is one of the top serovars causing human salmonellosis. The core genome single nucleotide variant pipeline (cgSNV) is one of several whole genome based sequence typing methods used for the laboratory investigation of foodborne pathogens. SNV detection using this method requires a reference genome. The purpose of this study was to investigate the impact of the choice of the reference genome on the cgSNV-informed phylogenetic clustering and inferred isolate relationships. We found that using a draft or closed genome of *S*. Heidelberg as reference did not impact the ability of the cgSNV methodology to differentiate among 145 *S*. Heidelberg isolates involved in foodborne outbreaks. We also found that using a distantly related genome such as *S*. Dublin as choice of reference led to a loss in resolution since some sporadic isolates were found to cluster together with outbreak isolates. In addition, the genetic distances between outbreak isolates as well as between outbreak and sporadic isolates were overall reduced when *S*. Dublin was used as the reference genome as opposed to *S*. Heidelberg.

## Introduction

Nontyphoidal *Salmonella* (NTS) *enterica* serovars are the most important causes of bacterial gastroenteritis. Among the NTS serovars, Heidelberg is ranked as the second and third most frequent serovar recovered from clinical cases in Québec and Canada respectively [1]. In Québec between 2004 and 2014, 23% of *S*. Heidelberg clinical isolates were from blood specimens, compared to 7% for *S*. *enterica* serovar Enteritidis and 5% for *S*. *enterica* serovar Typhimurium, suggesting an increased capacity of this serovar to cause invasive systemic disease [2].

Pulsefield gel electrophoresis (PFGE) has been the gold standard method used by PulseNet Canada (PNC) since the 1990s for the molecular typing of *Salmonella* during outbreak investigations. However, a major drawback with the use of PFGE in outbreak investigation is the low resolution power of this technique that is further exacerbated when applied to *S*. Heidelberg typing owing to the extremely low genetic diversity of this serovar. For example, 70% of *S*. Heidelberg isolated in Québec belonged to pulsovar 2 [2].

Whole genome sequence (WGS) based methods owing to their growing availability and high genomic resolution are rapidly replacing traditional typing methods such as PFGE within major public health laboratories including PNC [3]. Two popular methods that are increasingly applied in the field of bacterial genomic epidemiology are: the gene-by-gene methods which is basically an extension of the 7 gene MLST typing technique to encompass the entire genome (whole genome MLST, wgMLST) or just the core genome (core genome MLST, cgMLST) [4, 5] and the single nucleotide variant (SNV) methods which identifies single nucleotide variants by comparing a population of target genomes against a reference [6, 7]. We recently found that the cgSNV method provided superior discriminatory power than traditional methods during outbreak investigations involving *Salmonella* Heidelberg [2].

The choice of the reference genome has been previously proposed as a potential consideration affecting core genome SNV (cgSNV)–based analysis and outcomes. For example, choosing a distantly related strain as a source of reference may tend to cluster isolates that are otherwise genetically distant. Another concern with the choice of reference genome is the sequencing status of the reference genome. It is generally perceived that high-quality complete genomes are preferred to ensure accurate and epidemiologically concordant phylogenetic analysis and outbreak investigation. These concerns were not addressed in our previous work on the cgSNV method [2]. Here using draft *de novo* assembled and completely sequenced or closed genomes of *S*. Heidelberg as well as a distantly related genome such as *S*. Dublin as references, we assessed the ability of the cgSNV methodology to differentiate amongst 145 *S*. Heidelberg strains involved in four distinct outbreaks and sporadic cases of salmonellosis in Québec.

## Materials and method

### Collection and characterization of bacterial isolates

The 145 *S*. Heidelberg clinical isolates described in this study were collected as part of the Quebéc surveillance program on human salmonellosis established since 2003 to ensure rapid detection of outbreaks. The food isolates were collected by the Ministère de l'Agriculture, des Pêcheries et de l'Alimentation du Québec (MAPAQ) during routine food-poisoning investigations. Isolates were grown on triple sugar iron agar at 37°C and stored at -80°C in trypticase soy spiked with 10% glycerol. PFGE and serotyping was performed at the Laboratoire de Santé Publique du Québec (LSPQ) following PNC guidelines.

### Whole genome sequencing

Frozen bacterial isolates were cultured overnight at 37°C in brain heart infusion broth and genomic DNA was extracted using the Metagenomic DNA isolation Kit for Water (Epicentre, Madison, WI). Samples were prepared using Nextera XT chemistry (Illumina, Inc., San Diego, CA) and were sequenced using Illumina Miseq paired-end read technology using 300 base read lengths. Five strains were selected from the outbreak isolates to serve as references and their reads were *de novo* assembled using SPAdes v. 3.9 [8]. The complete genome counterparts for these strains have been reported in a previous work [9]. Two draft and three complete, unrelated reference genomes were also downloaded from NCBI and included in this analysis making a total of 15 assessed reference genomes (Table 1).

**Table 1:** General features of the strains used as reference genomes for the cgSNV analysis of 145 *S*. Heidelberg isolates.

Strain ID	Genome Status	NCBI Accession No	Source	Serovar	Genome used as reference in Tree number
ID117795	Draft	NA	Human	Heidelberg	1
ID117795	Completely sequenced	CP016507	Human	Heidelberg	2
ID128787	Draft	NA	Human	Heidelberg	3
ID128787	Completely sequenced	CP016586	Human	Heidelberg	4
ID128902	Draft	NA	Human	Heidelberg	5
ID128902	Completely sequenced	CP016579	Human	Heidelberg	6
ID134609	Draft	NA	Human	Heidelberg	7
ID134609	Completely sequenced	CP016581	Human	Heidelberg	8
ID135140	Draft	NA	Food	Heidelberg	9
ID135140	Completely sequenced	CP016510	Food	Heidelberg	10
SL486	Draft	NZ_ABEL00000000	Human	Heidelberg	11
CFSAN024776	Draft	NC_JWQE00000000	Human	Heidelberg	12
SL476	Completely sequenced	NC_011083	Ground turkey	Heidelberg	13
B182	Completely sequenced	CP003416	Bovine feces	Heidelberg	14
SL477	Completely sequenced	CP001144	Human	Dublin	NA

NA, Not available.

### CgSNV typing

CgSNV analysis was performed using the SNVPhyl pipeline [10] v.1.0 integrated within the NML instance of the Galaxy platform [11]. Briefly, paired-end sequence reads from the 145 isolates were aligned against each of the 15 reference genomes using SMALT v.0.7.5 (http://www.sanger.ac.uk/science/tools/smalt-0). MUMmer v.3.23 [12] and PHAST [13] were used to identify repeat and prophage regions in each reference genome respectively and these regions were excluded from the analysis. Variants were called using two independent variant calling algorithms: FreeBayes v.0.9.20 and SAMtools [14] /BCFtools based on predefined criteria described elsewhere [10]. To infer the relationship between these isolates, minimum spanning trees were constructed from the SNVphyl output data using the geoBURST algorithm built into PHYLOViZ v2.0 [15].

### Topological similarity

We assessed the topological similarity of the phylogenetic trees using the Robinson and Foulds (RF) test [16]. This test is a widely used tree metric for tree-to-tree distances and is defined as the minimum number of operations needed to transform one tree into the other. Briefly, newick tree files from the SNVphyl pipeline generated using each of the 14 genomes as references were concatenated and the resulting file was submitted to the online phylogenetic tool T-REX [17] to compute the topological distances between the trees.

### Nucleotide sequence accession numbers

The sequence data supporting the results of this article have been deposited in the NCBI Sequence Read Archive under accession number SRP098783.

## Results

### Epidemiological characteristics and PFGE subtyping results of the 145 *S*. Heidelberg isolates

Epidemiologic and PFGE fingerprinting results of the 145 S. Heidelberg isolates used in this study are presented in Table 2.

**Table 2:** Epidemiologic and subtyping results of the 145 S. Heidelberg clinical and food isolates used in this study.

Isolate No.	Source	Isolation date	Outbreak code	Pulsotype	Phage type	NCBI accession no.
ID117793	Human	05–2012	1	2	19	SH12-001
ID117794	Human	05–2012	1	2	19	SH12-002
ID117795	Human	05–2012	1	2	19	SH12-003
ID117796	Human	05–2012	1	2	19	SH12-004
ID117797	Human	05–2012	1	2	19	SH12-005
ID117798	Human	05–2012	1	2	19	SH12-006
ID117799	Human	05–2012	1	2	19	SH12-007
ID117800	Human	05–2012	1	2	19	SH12-008
ID118040	Food	05–2012	1	2	19	SH12-009
ID117870	Food	05–2012	1	2	19	SH12-010
ID128696	Human	11–2013	2	2	26	SH13-001
ID128783	Human	11–2013	2	2	26	SH13-002
ID128786	Human	11–2013	2	2	26	SH13-003
ID128787	Human	11–2013	2	2	26	SH13-004
ID128808	Human	11–2013	2	2	26	SH13-005
ID128902	Human	11–2013	2	2	26	SH13-006
ID128908	Human	11–2013	2	2	26	SH13-007
ID128910	Human	11–2013	2	2	26	SH13-008
ID134557	Human	07–2014	3	2	19	SH14-001
ID134930	Human	08–2014	3	2	19	SH14-002
ID134612	Human	08–2014	3	2	19	SH14-003
ID134421	Human	08–2014	3	2	19	SH14-004
ID134719	Human	08–2014	3	2	19	SH14-005
ID134608	Human	08–2014	3	2	19	SH14-006
ID135122	Human	08–2014	3	2	19	SH14-007
ID134610	Human	08–2014	3	2	19	SH14-008
ID134609	Human	08–2014	3	2	19	SH14-009
ID134565	Human	08–2014	3	2	17	SH14-010
ID134559	Human	08–2014	3	2	17	SH14-011
ID134929	Human	08–2014	3	2	ATHE-35	SH14-012
ID134879	Food	08–2014	3	2	19	SH14-013
ID134880	Food	08–2014	3	2	19	SH14-014
ID134881	Food	08–2014	3	2	19	SH14-015
ID134882	Food	08–2014	3	2	19	SH14-016
ID134883	Food	08–2014	3	2	19	SH14-017
ID134884	Food	08–2014	3	2	19	SH14-018
ID134885	Food	08–2014	3	2	19	SH14-019
ID134886	Food	08–2014	3	2	19	SH14-020
ID134887	Food	08–2014	3	2	19	SH14-021
ID134888	Food	08–2014	3	2	19	SH14-022
ID134889	Food	08–2014	3	2	19	SH14-023
ID134890	Food	08–2014	3	2	19	SH14-024
ID135137	Food	08–2014	3	2	19	SH14-025
ID135138	Food	08–2014	3	2	19	SH14-026
ID135139	Food	08–2014	3	2	19	SH14-027
ID135140	Food	08–2014	3	2	19	SH14-028
ID148030	Human	03–2016	4	2	19	SRR5228105
ID148149	Human	03–2016	4	2	19	SRR5228097
ID148230	Human	03–2016	4	2	19	SRR5228082
ID148231	Human	03–2016	4	2	19	SRR5228079
ID148280	Human	03–2016	4	2	19	SRR5228104
ID148286	Human	03–2016	4	2	19	SRR5228087
ID148337	Human	03–2016	4	2	19	SRR5228078
ID148338	Human	03–2016	4	2	19	SRR5228091
ID094525	Human	12–2007	NA	2	19	SRR5227118
ID095996	Human	04–2008	NA	3	11	SRR5227171
ID097320	Human	07–2008	NA	2	19	SRR5227121
ID099254	Human	10–2008	NA	2	29	SRR5227124
ID099787	Human	12–2008	NA	2	19	SRR5228101
ID100344	Human	01–2009	NA	2	19	SRR5227119
ID100753	Human	02–2009	NA	2	19	SRR5227155
ID101488	Human	04–2009	NA	122	16	SRR5227148
ID102666	Human	07–2009	NA	2	26	SRR5227120
ID102743	Human	08–2009	NA	2	19	SRR5227166
ID102860	Human	08–2009	NA	17	19	SRR5227126
ID102963	Human	08–2009	NA	2	26	SRR5228093
ID103472	Human	09–2009	NA	2	19	SRR5227163
ID103849	Human	10–2009	NA	1	2	SRR5227117
ID103978	Human	10–2009	NA	138	16	SRR5227128
ID104279	Human	11–2009	NA	140	1	SRR5227146
ID104398	Human	12–2009	NA	1	2	SRR5227169
ID105089	Human	02–2010	NA	6	32	SRR5227122
ID105144	Human	02–2010	NA	2	19	SRR5227127
ID106827	Human	06–2010	NA	87	32	SH12-013
ID107176	Human	07–2010	NA	2	29	SRR5228100
ID107454	Human	07–2010	NA	1	2	SRR5227152
ID108191	Human	08–2010	NA	2	19	SH10-001
ID108221	Human	08–2010	NA	2	26	SH10-014
ID108759	Human	09–2010	NA	86	26	SRR5227162
ID108677	Human	09–2010	NA	2	26	SH10-015
ID110275	Human	01–2011	NA	165	35	SRR5227139
ID110331	Human	01–2011	NA	107	22	SRR5227156
ID110403	Human	01–2011	NA	2	19	SRR5227141
ID110674	Human	02–2011	NA	168	atypical	SRR5227174
ID110801	Human	02–2011	NA	2	26	SH11-002
ID111466	Human	04–2011	NA	2	19	SRR5227167
ID113160	Human	08–2011	NA	66	29	SRR5227130
ID113273	Human	08–2011	NA	175	47	SRR5227140
ID113787	Human	09–2011	NA	178	atypical	SRR5227173
ID114520	Human	10–2011	NA	2	29	SRR5227135
ID114593	Human	11–2011	NA	2	29	SRR5227157
ID115377	Human	12–2011	NA	2	19	SRR5227165
ID115568	Human	01–2012	NA	86	29	SRR5227133
ID116136	Human	02–2012	NA	2	29	SRR5227164
ID116271	Human	02–2012	NA	87	32	SH10-014
ID116824	Human	03–2012	NA	107	ATHE-10	SRR5227129
ID117211	Human	04–2012	NA	2	29	SRR5227151
ID117095	Human	04–2012	NA	2	29	SRR5227172
ID117506	Human	04–2012	NA	2	19	SH10-002
ID117683	Human	04–2012	NA	2	19	SRR5227143
ID117578	Human	05–2012	NA	2	19	SH12-011
ID118209	Human	05–2012	NA	4	5	SRR5227123
ID118236	Human	05–2012	NA	86	29	SRR5227154
ID118280	Human	05–2012	NA	107	ATHE-10	SRR5227150
ID118551	Human	06–2012	NA	52	10	SRR5227136
ID118532	Human	06–2012	NA	52	10	SRR5227175
ID118700	Human	06–2012	NA	52	10	SRR5227158
ID118759	Human	06–2012	NA	2	18	SH12-012
ID118979	Human	07–2012	NA	2	19	SRR5227159
ID119224	Human	07–2012	NA	2	29	SRR5227125
ID119366	Human	07–2012	NA	186	10	SRR5227138
ID119464	Human	08–2012	NA	2	17	SRR5227145
ID119539	Human	08–2012	NA	2	19	SRR5227147
ID119674	Human	08–2012	NA	2	19	SRR5227170
ID119888	Human	08–2012	NA	52	10	SRR5227161
ID119967	Human	08–2012	NA	2	19	SRR5227144
ID120403	Human	09–2012	NA	2	19	SRR5227160
ID120598	Human	09–2012	NA	2	19	SRR5227132
ID120747	Human	09–2012	NA	2	19	SRR5227131
ID121956	Human	11–2012	NA	189	10	SRR5227149
ID122356	Human	12–2012	NA	2	19	SRR5227176
ID124024	Human	03–2013	NA	2	19	SRR5227168
ID124305	Human	04–2013	NA	2	29	SRR5227153
ID124498	Human	04–2013	NA	4	35	SRR5227142
ID125378	Human	06–2013	NA	2	19	SRR5227137
ID126392	Human	07–2013	NA	2	19	SRR5227134
ID126712	Human	08–2013	NA	2	19	SRR5228099
ID126777	Human	08–2013	NA	2	19	SRR5228083
ID126776	Human	08–2013	NA	2	19	SRR5228080
ID126696	Human	08–2013	NA	2	19	SRR5228081
ID126825	Human	08–2013	NA	2	29	SRR5228085
ID147047	Human	02–2016	NA	4	5	SRR5228102
ID147120	Human	02–2016	NA	231	32	SRR5228084
ID147091	Human	02–2016	NA	52	10	SRR5228088
ID147129	Human	02–2016	NA	2	19	SRR5228092
ID147253	Human	02–2016	NA	194	29	SRR5228086
ID147255	Human	02–2016	NA	229	10	SRR5228090
ID147457	Human	02–2016	NA	225	19	SRR5228089
ID147462	Human	02–2016	NA	214	29	SRR5228094
ID147990	Human	03–2016	NA	52	10	SRR5228106
ID147796	Human	03–2016	NA	52	10	SRR5228095
ID147816	Human	03–2016	NA	2	19	SRR5228103
ID147910	Human	03–2016	NA	214	29	SRR5228077
ID147841	Human	03–2016	NA	2	19	SRR5228096
ID148066	Human	03–2016	NA	2	19	SRR5228098

NA, Not available.

Isolates from four distinct outbreaks that occurred in Quebéc between 2012 and 2016 were also included in the analysis. These outbreaks were designated as follows: outbreak 1, 2012 (n = 10; 8 human and 2 food isolates) outbreak 2, 2013 (n = 8 human isolates) outbreak 3, 2014 (n = 28; 12 human and 16 food isolates and outbreak 4, 2016 (n = 8 human isolates). All human cases and food items linked to these outbreaks were confirmed by epi-data. Outbreak 1 was linked to a wedding party, outbreak 2 and 3 were traced to separate restaurants and outbreak 4 was associated with a daycare catering service. In addition to the outbreak isolates (n = 54) we also added 91 sporadic clinical isolates collected in Quebéc between 2007 and 2016 into the analysis.

### Whole genome sequencing results

An average of 983,919 reads was obtained per isolate (range, 339,270–2,974,917) for the set of 145 *S*. Heidelberg isolates, corresponding to an estimated average genome coverage of 121x (range, 42x -365x). The number of SPAdes-assembled contigs for the five outbreak isolates that were selected to act as the reference ranged from 24–27 with all the isolates assembled into fewer than 27 contigs (Table 3). The completely sequenced genome equivalent of these isolates have been published in a previous study [9].

**Table 3:** Assembly statistics for the 5 *S*. Heidelberg isolates that served as reference genomes.

Strain	Total length (bp)	No of contigs	N50 (bp)	Coverage
ID117795	4,751,241	24	694,16	137
ID128787	4,747,971	27	363,273	43
ID128902	4,746,565	27	412,159	64
ID134609	4,853,519	27	412,096	130
ID135140	4,753,550	27	412,162	116

### Core genome single nucleotide analysis

**Fig 1:**
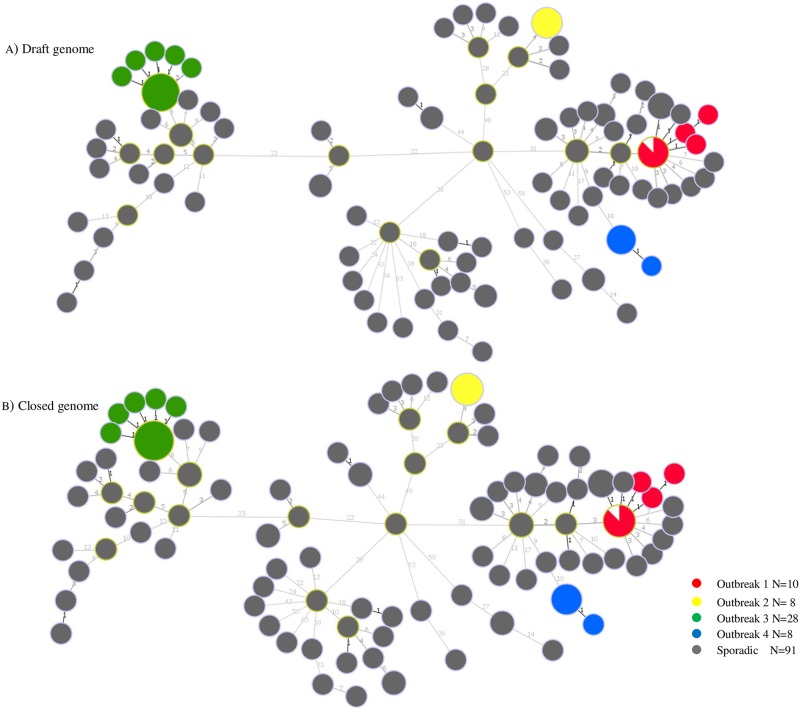
Minimum spanning phylogenetic tree of the core genome of 145 S. Heidelberg sequenced isolates generated using A) draft genome or B) closed referenced genome (ID117795) as an example. Isolates in the same circle have 0 hqSNVs and the size of each circle is proportional to the number of isolates in the circle.

After removing repeats and prophages as well as SNV-dense regions from all the reference genomes, an average of 4,008,254 genomic positions (range 3,681,444–4,049,343) representing an average of 86% of the reference genomes (range 84.63–86.72%) had sufficient coverage (≥15x) across all 145 isolates for reference mapping. For all the *S*. Heidelberg reference genomes, an average of 769 high-quality consensus SNVs (range 751–819) were identified by both variant callers as common to all isolates and used for subsequent phylogenetic clustering. For the distantly related *S*. Dublin genome, 18,155 high-quality core genome SNVs were used to construct the phylogeny. In total, 15 minimum spanning trees were generated with 14 of these trees representing the 14 *S*. Heidelberg reference genomes. All outbreak isolates formed distinct clusters with all the 14 *S*. Heidelberg reference genomes and the topologies of these trees were highly similar (Fig 1A and 1B).

**Fig 2:**
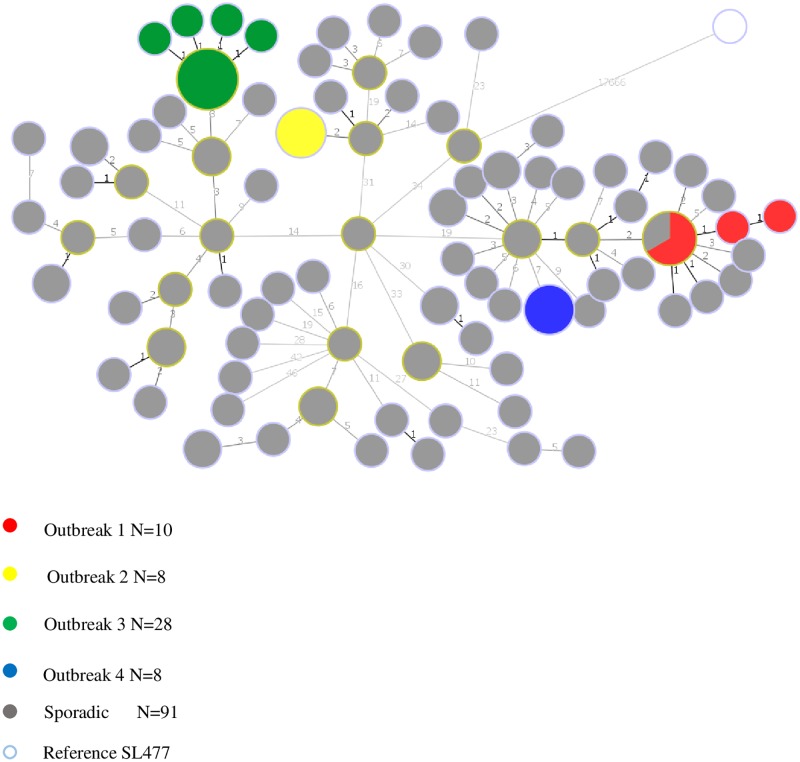
Minimum spanning phylogenetic tree of the core genome of 145 *S*. Heidelberg sequenced isolates generated using the distantly related reference *S*. Derby (SL477). Isolates in the same circle have 0 hqSNVs and the size of each circle is proportional to the number of isolates in the circle.

Using *S*. Dublin as the reference genome led to a loss in resolving power. In fact, sporadic isolates clustered with outbreak 1 isolates (Fig 2).

The phylogenetic features revealed by the minimum spanning trees were nearly identical across all the 14 *S*. Heidelberg reference genomes (Table 4).

**Table 4:** Phylogenetic observations of the minimum spanning tree topology built from cgSNV analysis of 145 *S*. Heidelberg isolates and comparison of draft and closed genomes as references.

References selected from within the outbreak isolates											References downloaded from NCBI				Distantly related genome downloaded from NCBI
Minimum spanning tree features	ID117795		ID128787		ID128902		ID134609		ID135140		SL486	JWQE01.1	SL476	CP003416.1	CP001144
	Draft	Closed	Draft	Closed	Draft	Closed	Draft	Closed	Draft	Closed	Draft	Draft	Closed	Closed	Closed
Total # of nodes on the tree	92	92	92	92	92	92	93	93	93	93	92	93	93	93	86
Total # of nodes representing 1 isolate	79	79	79	79	79	79	80	80	80	80	78	79	80	80	72
Total # of nodes with more than one isolate	13	13	13	13	13	13	13	13	13	13	13	14	13	13	15
Total # of OB nodes	13	13	13	13	13	13	13	13	13	13	13	13	13	13	10
Total # of nodes with one isolate	9	9	9	9	9	9	9	9	9	9	9	9	9	9	6
Total # of OB nodes with more than one isolate	4	4	4	4	4	4	4	4	4	4	4	4	4	4	4
Total # of sporadic nodes	79	79	79	79	79	79	80	80	80	80	78	79	80	80	76
Total # of sporadic nodes linked to main OB clusters	10	10	10	10	10	10	10	10	10	10	10	10	10	10	10
Number of sites used to generate phylogeny	762	762	761	760	762	760	766	761	764	763	777	799	751	819	18155
Number of genomic positions used for reference mapping	4,046,580	4,037,652	4,046,584	4,037,362	4,047,586	4,037,102	4,049,343	4,037,849	4,046,484	4,037,788	4,008,004	4,048,063	3,925,643	4,036,335	3,681,444
% of genomic positions representing the genome	86,04%	86,68%	86,07%	86,7%	86,08%	86,69%	85%	86,72%	86%	86,72%	85,95%	86,12%	85,72%	86,67%	84,63%

The genetic distances observed between outbreak isolates as well as between outbreak and sporadic isolates were nearly identical across the 14 *S*. Heidelberg reference genomes. Using *S*. Dublin as the reference genome led to a significant reduction in genetic distances between sporadic and outbreak isolates with some sporadic isolates having 0 and 3 SNV difference with outbreak 1 and 3 respectively whereas the genetic distances between isolates in these outbreaks ranged from 0–3 SNVs (Table 5).

**Table 5:** Comparison of the number of high quality SNVs between 145 *S*. Heidelberg sporadic and outbreak isolates using a draft, closed and distantly related reference genomes.

Reference^a^	Outbreak	Outbreak 1		Outbreak 2		Outbreak 3		Outbreak 4		Sporadic	
		Draft	Closed	Draft	Closed	Draft	Closed	Draft	Closed	Draft	Closed
**ID117795**	**1**	0–3	0–3	72–74	71–73	48–51	48–52	18–21	18–21	1–93	1–93
	**2**	72–74	71–73	0	0	66–67	65–67	80–81	79–80	4–105	4–104
	**3**	48–51	48–52	66–67	65–67	0–2	0–3	56–58	56–59	6–86	6–87
	**4**	18–21	18–21	80–81	79–80	56–58	56–59	0–1	0–1	10–100	10–100
**ID128787**	**1**	0–3	0–3	71–73	71–73	47–50	47–50	17–20	17–20	1–92	1–92
	**2**	71–73	71–73	0	0	66–67	66–67	80–81	80–81	4–105	4–105
	**3**	47–50	47–50	66–67	66–67	0–2	0–2	56–58	56–58	6–86	6–86
	**4**	17–20	17–20	80–81	80–81	56–58	56–58	0–1	0–1	10–100	10–100
**ID128902**	**1**	0–3	0–3	72–74	71–73	48–52	47–50	17–20	17–20	1–92	1–92
	**2**	72–74	71–73	0	0	66–68	66–67	81–82	80–81	4–105	4–105
	**3**	48–52	47–50	66–68	66–67	0–3	0–2	57–60	56–58	6–86	6–86
	**4**	17–20	17–20	81–82	80–81	57–60	56–58	0–1	0–1	10–101	10–100
**ID135140**	**1**	0–3	0–3	72–74	71–73	48–52	47–51	17–20	17–20	1–93	1–92
	**2**	72–74	71–73	0	0	66–68	66–68	81–82	80–81	4–105	4–105
	**3**	48–52	47–51	66–68	66–68	0–3	0–3	58–60	58–59	6–87	6–87
	**4**	17–20	17–20	81–82	80–81	58–60	58–59	0–1	0–1	10–100	10–100
**ID135609**	**1**	0–3	0–3	72–74	71–73	48–52	47–50	17–20	17–20	1–93	1–92
	**2**	72–74	71–73	0	0	66–68	66–67	81–82	80–81	4–105	4–105
	**3**	48–52	47–50	66–68	66–67	0–3	0–2	57–60	56–58	6–86	6–86
	**4**	17–20	17–20	81–82	80–81	57–60	56–58	0–1	0–1	10–101	10–100
**SL486**	**1**	0–3	ND	71–73	ND	46–49	ND	17–20	ND	1–92	ND
	**2**	71–73		0		65–66		80–81		4–105	
	**3**	46–49		65–66		0–2		55–57		6–85	
	**4**	17–20		80–81		55–57		0–1		13–100	
**JWQE01.1**	**1**	0–3	ND	71–73	ND	46–49	ND	17–20	ND	1–91	ND
	**2**	70–72		0		66–67		79–80		4–105	
	**3**	46–49		66–67		0–2		55–57		6–86	
	**4**	17–20		79–80		55–57		0–1		10–99	
**SL476**	**1**	ND	0–3	ND	68–70	ND	46–49	ND	17–20	ND	1–92
	**2**		68–70		0		65–66		80–81		4–105
	**3**		46–49		65–66		0–2		55–57		6–85
	**4**		17–20		80–81		55–57		0–1		13–100
**CP003416.1**	**1**	ND	0–3	ND	72–74	ND	47–50	ND	17–20	ND	1–92
	**2**		72–74		0		67–68		81–82		4–106
	**3**		47–50		67–68		0–2		56–58		6–86
	**4**		17–20		81–82		56–58		0–1		10–100
**CP001144**	**1**	ND	0–2	ND	45–47	ND	28–31	ND	10–12	ND	0–60
	**2**		45–47		0		39–40		49–49		2–67
	**3**		28–31		39–40		0–2		32–34		3–53
	**4**		10–12		49–49		32–34		0		7–62

^a^The reference genomes were obtained from: *S*. Heidelberg outbreak isolates [ID117795, ID128787, ID128902, ID135140, and ID135609]; publicly-available *S*. Heidelberg references from NCBI [SL486, JWQE01.1, SL476, CP003416.1] and distantly-related *S*. Dublin reference from NCBI [CP001144].

ND, Not Determined.

### Topological similarity of the phylogenetic trees

To confirm the similarities in tree topologies, we performed the RF test. The computed RF topological distances between the 14 trees ranged from 0 to 24 (Table 6).

**Table 6:** Robinson-Foulds topological distances between trees generated with 145 *S*. Heidelberg sequenced isolates using draft and closed genomes as references during cgSNV analysis.

	Tree 2	Tree 3	Tree 4	Tree 5	Tree 6	Tree 7	Tree 8	Tree 9	Tree 10	Tree 11	Tree 12	Tree 13	Tree 14
Tree 1	0	20	20	20	20	20	20	20	20	7	11	15	11
Tree 2		20	20	20	20	20	20	20	10	7	11	15	11
Tree 3			0	0	0	24	0	24	24	13	11	15	11
Tree 4				0	0	24	0	24	24	13	11	15	11
Tree 5					0	24	0	24	24	13	11	15	11
Tree 6						24	0	24	24	13	11	15	11
Tree 7							24	0	0	13	15	19	15
Tree 8								24	24	13	11	15	11
Tree 9									0	13	15	19	15
Tree 10										13	15	19	15
Tree 11											4	8	4
Tree 12												4	0
Tree 13													4

## Discussion

In this study we assessed the impact of the choice of the reference genome on the resolution clustering of *S*. Heidelberg outbreak and sporadic isolates using the cgSNV methodology. Our results revealed that using a draft or completely sequenced *S*. Heidelberg genomes as references did not affect the ability of the cgSNV method to distinguish between four epidemiologically well characterized *S*. Heidelberg outbreak isolates and to separate these isolates from sporadic or background strains. In fact, all outbreak and sporadic isolates were clustered on distinct branches (Fig 1A and 1B). On the contrary, using *S*. Dublin as reference choice resulted in a tree with less resolution (Fig 2). Sporadic isolates clustered together with outbreak 1 isolates and in addition, the genetic distances observed within outbreak as well as between outbreak and sporadic isolates was overall reduced when *S*. Dublin was used as reference choice as opposed to *S*. Heidelberg reference genomes (Figs 1 and 2, Table 5). This finding is in agreement with a recent study on *Salmonella* which found that using a distantly related genome as a choice of reference failed to cluster *S*. Enteritidis outbreak strains concordantly [18]. These observations emphasize the need to choose an appropriate reference genome during laboratory investigations of foodborne outbreaks involving reference based methods.

The loss in resolution observed in this study can be due to the following reasons: Firstly, the SNVPhyl pipeline uses only core genome SNVs to build the phylogeny implying that a reference genome with high similarity with the isolates under investigation would have a larger core genome from which more SNVs can be produced to build a phylogeny as opposed to a dissimilar reference such as *S*. Dublin. However, the smallest core genome among all the reference genomes used in this study was equivalent to 84.76% of the total genome. Secondly, another issue to consider is that as the reference genome grows more distant from the sequences under analysis, more variation would be observed between the core regions of the reference genome and all other sequences leading to a long branch separating the reference genome from the rest of the other isolates. This was indeed what we observed using *S*. Dublin as reference (Fig 2) since the majority of the 18155 positions used to construct the phylogeny were indeed variations between the reference (17666 positions) and the other isolates.

The RF values for the trees constructed using *S*. Heidelberg draft genome and its corresponding closed genome equivalent was zero with the exception of the reference genome pair ID134609 (tree 7 vs. tree 8) whereby the RF topological distance was 24. This difference could be attributed to misidentification of SNVs linked to the presence of repetitive regions in the draft genome that were not properly detected. By nature, draft genomes are not entirely genomically accurate and for this reason, it is possible that the SPAdes assembly for this isolate may have collapsed repetitive regions larger than the read/read pair size into a single contig. Aligning reads to repetitive regions is problematic and has been reported to lead to potential misidentification of SNVs [19]. Although we used the software MUMmer to identify and filter the repetitive regions of the reference genome, for a closed genome, this method will be much more successful due to its intact, completely mapped sequence than for a draft genome, which may erroneously possess several copies of unmapped repeat regions. These unmapped repeats can lead to repeat reads mapping to a single contig resulting in the inclusion of additional SNVs and subsequent alignment issues and false SNV calls. Interestingly in this reference pair, 766 sites were used to generate the phylogeny using the draft genome as reference as opposed to 761 sites for the closed genome counterpart. Despite the slight differences in topology between this reference pair as well as in other tree pairs, both the draft and closed genomes were still able to differentiate the outbreak from sporadic isolates in concordance with epidemiological data. In agreement with our observations, a recent study on *Listeria monocytogenes* also demonstrated that phylogenetic clustering based on SNV analysis using a *de novo* assembled draft genome selected from within the group was similar to the phylogeny using a closely related closed genome [6].

Despite the increasing accessibility of WGS-based technologies and decreasing costs, the implementation of WGS for routine surveillance and outbreak investigation may still present many challenges as sequencing a genome to completion remains a costly and time-consuming endeavour and for many public-health laboratories, this is not a viable option. [20]. The results of our study indicates that draft genomes can be relied upon as a suitable reference choice during laboratory investigations of foodborne outbreaks using the cgSNVphyl pipeline. In fact, a recent study evaluated the quality score of 32000 genomes located in public repositories and concluded that most of these genomes were of sufficient quality to perform analysis on and only 10% of draft genomes were of poor quality and unsuitable for downstream analysis [21]. In conclusion, our results provide strong evidence that the choice of the reference genome does not impact the ability of the cgSNV methodology to distinguish between *S*. Heidelberg isolates involved in foodborne outbreaks. Our results also demonstrate that using a distantly related genome as reference could lead to a loss in resolution during cgSNV analysis. Although wgMLST was recently recommended as the primary subtyping tool moving forward by PNC and other foodborne surveillance networks [22], it is important to note that the cgSNV approach still remains an important method in the PNC molecular tool box. In fact, this method was recently used by PNC in collaboration with provincial public health laboratories to identify the source of a multi-provincial outbreak of *S*. Enteritidis [23]. Despite the advantages provided by cg/wgMLST approaches such as curability and standardization, the development and validation of schemas for each organism still remains a daunting task both financially and technically for public health laboratories operating with limited resources. The implementation of the cgSNV methodology described here could be a viable alternative for monitoring *S*. Heidelberg. Whether this method applies to other *Salmonella* serovars remains to be determined.
